# Structural overview of lyssavirus glycoproteins, antibodies, and receptors

**DOI:** 10.1128/jvi.01786-25

**Published:** 2026-06-02

**Authors:** Heather M. Callaway

**Affiliations:** 1 Department of Chemistry and Biochemistry, Montana State University 33052, Bozeman, Montana, USA; 2 Department of Microbiology and Cell Biology, Montana State University 33052, Bozeman, Montana, USA; Indiana University Bloomington, Bloomington, Indiana, USA

**Keywords:** rabies, lyssaviruses, receptor binding, neutralizing antibodies, structures, structural biology

## Abstract

Rabies virus is the most lethal virus ever discovered and remains a global health threat despite vaccines and post-exposure treatment. In addition to rabies, there are also 17 other lyssaviruses, several of which have already crossed species barriers to infect humans and cause the same clinical disease as the rabies virus. While effective in preventing rabies infection, current rabies vaccines do not provide long-lasting protection or elicit antibodies that are broadly protective against both rabies and related lyssaviruses. Efforts to improve rabies vaccines to elicit a uniform, longer-lasting, and more broadly neutralizing antibody response would benefit from structure-guided design, where high-resolution protein structures are used to engineer vaccine antigens. In the last 6 years, the first high-resolution structures of lyssavirus glycoproteins have become available, giving new insights into how these viruses interact with host antibodies and receptors, and making structure-guided antigen design feasible. This review encompasses recent findings in lyssavirus glycoprotein structure, interactions with neutralizing antibodies, and interactions with potential cellular receptors, with an emphasis on the rabies virus.

## INTRODUCTION

Rabies is the most lethal virus ever discovered, with a nearly 100% fatality rate. Upward of 59,000 people die from untreated rabies cases each year, with 40% of those deaths occurring in children (1, 2). While lifesaving vaccines and a post-exposure treatment exist for the rabies virus, neither the current vaccines nor the post-exposure treatment offer long-lasting protection or are effective in patients who have already developed symptoms of infection (3–5). Furthermore, polyclonal antibodies induced by rabies vaccines, also used in rabies post-exposure treatment, do not effectively neutralize non-rabies lyssaviruses (6). Seventeen of these non-rabies lyssaviruses have been discovered (7, 8), and several have already crossed species barriers to infect humans (7) and kill with the same clinical disease as rabies virus (7, 9–12).

Most human rabies exposures and deaths result from dog bites, especially in parts of the world where feral dogs are common, animal rabies vaccination programs are limited, and human rabies post-exposure treatment is either unaffordable or limited in availability (2). In the United States, Europe, and other countries where veterinary rabies vaccination programs are extensive and rabies post-exposure treatment is both affordable and readily available, wildlife transmission (especially from bats) is instead the major means of rabies exposure and death (1). However, despite the lower numbers of deaths in these countries, rabies remains a large public health concern and economic burden. In the United States alone, 1.4 million people per year receive treatment for a possible rabies exposure (13), and rabies prevention and treatment are estimated to cost hundreds of millions of U.S. dollars per year (2, 14). Those costs range into multiple billions of dollars globally (2).

To prevent human rabies deaths, reduce the enormous economic burden of rabies prevention and treatment, and prepare for the possibility of future pandemics of rabies-related lyssaviruses, there is an urgent need to develop improved rabies vaccines and therapeutics. An ideal rabies vaccine would elicit a long-lasting, potent, and broadly neutralizing antibody response against rabies and multiple other lyssaviruses. Similarly, ideal rabies therapeutics, either antibodies or antiviral drugs, would be low-cost, effective against both rabies and related lyssaviruses, and effective in patients who have already developed symptoms of infection. However, developing these new vaccines and therapeutics will require a greater understanding of the structure of lyssaviruses and their surface glycoproteins, the target of the neutralizing antibody response. This review encompasses the structure and function of lyssavirus glycoproteins, including their roles in both the neutralizing antibody response and infection (binding/uptake and fusion), with an emphasis on rabies virus glycoprotein (RABV-G).

## LYSSAVIRUS GLYCOPROTEINS ARE ESSENTIAL FOR INFECTION AND VIRAL NEUTRALIZATION

Lyssaviruses are bullet-shaped, enveloped viruses that encode a total of five proteins: the viral glycoprotein (G), the nucleocapsid protein (N), the matrix protein (M), the phosphoprotein (P), and the RNA-dependent RNA polymerase (L). The viral glycoprotein is the only protein on the surface of rabies and other lyssaviruses, and is responsible for both receptor binding and fusion of the viral and cell membranes. The glycoprotein is also the sole target of the neutralizing antibody response, the established correlate of protection against rabies infection (3).

Lyssavirus glycoproteins are type I transmembrane proteins and type III viral fusion proteins. They consist of a 19-amino-acid N-terminal signal peptide that is cleaved in the mature protein, a large N-terminal extracellular domain (residues 20–458; 1–439 if signal peptide is excluded), a transmembrane domain (residues 459–481; 440–462 without signal peptide), and a short cytoplasmic domain (residues 482–524; 463–505 without signal peptide) (15, 16). The cytoplasmic domain is reported to interact with the viral matrix protein (17) and is highly divergent between different rabies virus strains (~67% conserved when comparing CVS to PV rabies, for example).

Prior to 2020, no structure of any lyssavirus glycoprotein, partial or whole, had been determined. However, in the last 6 years, crystal structures of rabies virus glycoprotein (RABV-G) have been determined as a pre-fusion monomer (18) and a post-fusion monomer (18), and cryo-EM studies have yielded the structure of the pre-fusion trimer (19–21). In addition to RABV-G, structures have also been determined for the Mokola lyssavirus glycoprotein (MOKV-G) as a post-fusion monomer (22, 23) and for the Ikoma lyssavirus glycoprotein (IKOV-G) pre-fusion trimer (23). Several of these structures have also been solved in complex with neutralizing monoclonal antibodies (18–21, 23–25), as described in detail below. These studies, in combination with prior biochemical assays and negative stain electron microscopy, reveal the overall structure of lyssavirus glycoproteins and give context to prior work on antibody and receptor binding sites, previously identified largely by mutagenesis and competition (6, 15, 26–28).

Lyssavirus glycoproteins reversibly transition between pre-fusion, post-fusion, monomeric, and trimeric conformations (29–32). This transition is largely pH-driven, with acidic pH (~pH 5.9 or below, depending on strain) shifting the conformational equilibrium towards the post-fusion state and triggering fusion (29). Even at pH values as low as 4.0, however, a proportion of RABV-G can be detected in the pre-fusion conformation (32). Similarly, at neutral pH, post-fusion glycoproteins can also be detected on the viral surface (32). As a result of this conformational heterogeneity, the surface of rabies virus appears to be a fuzzy outer layer with glycoproteins in multiple different conformations (30, 33, 34), rather than the uniform array sometimes seen in vesicular stomatitis virus (VSV) (35), a bullet-shaped rhabdovirus in the same viral family as the lyssaviruses. One consequence of this conformational heterogeneity is that neutralizing antibodies can recognize multiple different glycoprotein conformations, including pre-fusion, post-fusion, or both pre- and post-fusion conformations (18, 19, 21, 25, 36). However, that same heterogeneity complicates improved vaccine design. It is currently unknown which glycoprotein conformation or conformational intermediate elicits the most robust neutralizing antibody response. Furthermore, conformational heterogeneity of RABV-G in rabies vaccines may contribute to the large variations in neutralizing antibody titer observed between individuals (3) and may also reduce the magnitude of the antibody response, which is enhanced by multivalent, repeated epitopes during immunization (37).

## HIGH-RESOLUTION STRUCTURES OF LYSSAVIRUS GLYCOPROTEINS

**Fig 1:**
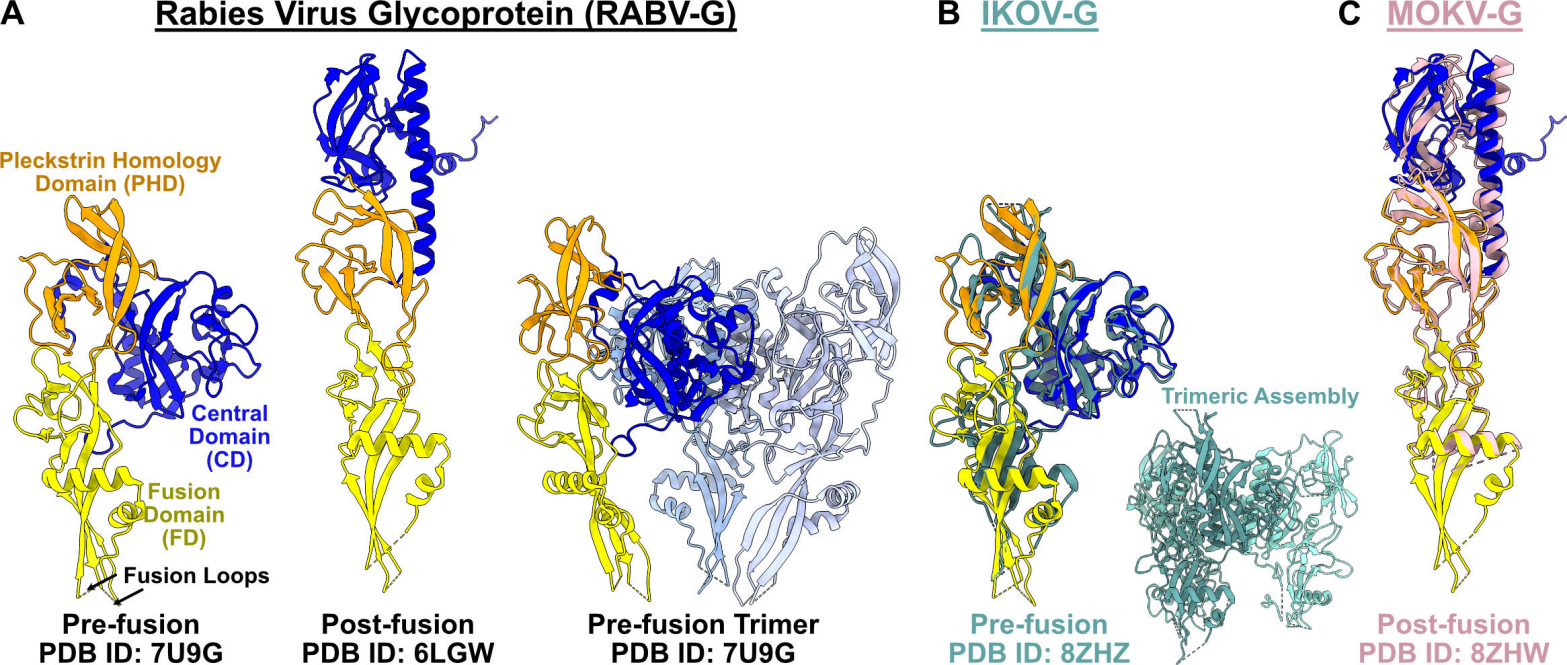
Lyssavirus glycoproteins share substantial structural similarity despite sequence variation. (**A**) Structures of RABV-G in the pre-fusion, post-fusion, and pre-fusion trimeric conformations, with domains color-coded and the fusion loops indicated with arrows. Alignments of Ikoma lyssavirus glycoprotein (**B**, teal) and Mokola lyssavirus glycoprotein (**C**, rose) are shown in combination with RABV-G pre-fusion and post-fusion glycoproteins, respectively.

High-resolution structures of lyssavirus glycoproteins have revealed a structural homology conserved across the lyssavirus genus and the rhabdovirus family despite substantial differences in amino acid sequence (18–20, 22, 23). The ectodomains of RABV-G, MOKV-G, IKOV-G, as well as the VSV glycoprotein, are ~440 amino acids long and contain three domains: the central domain, the fusion domain, and the Pleckstrin homology domain (Fig. 1) (18, 19, 21, 22, 38, 39). The central domain (CD) contains a long central helix and the trimeric interface of the glycoprotein. The fusion domain contains two short fusion loops with highly conserved aromatic amino acids that are essential for fusion. Finally, the Pleckstrin homology domain connects the central and fusion domains.

A conserved histidine residue between the central and Pleckstrin homology domains (H261 in RABV-G) triggers the pre-fusion to post-fusion transition (18, 20). Specifically, when that histidine is protonated as pH decreases, it may disrupt hydrogen bonding with an aspartic acid residue (D368) on an adjacent protomer (19), in turn destabilizing the pre-fusion trimer and facilitating a conformational change. During the transition to post-fusion, the central domain helix elongates and rearranges, substantially shifting the structure of the domain (Fig. 1) (18). In contrast, the fusion domain and Pleckstrin homology domain maintain their overall structure but shift their orientation relative to the viral membrane (18). In the pre-fusion conformation, the fusion loops at the bottom of the fusion domain point towards the viral membrane, while in the post-fusion conformation they rotate 180° outward to engage with a cellular membrane.

## GLYCOPROTEIN TRIMERIZATION

Like the pre-fusion to post-fusion transition, lyssavirus glycoprotein trimerization is also reversible. However, because RABV-G trimers are unstable with binding affinities for soluble ectodomains on the order of 10^−8^ M (19), solving their structures has proven challenging. No structures of any post-fusion lyssavirus glycoprotein trimer have yet been determined, but structures of the RABV-G and Ikoma-G pre-fusion trimers were recently solved (19, 20, 23). For the RABV-G pre-fusion trimer, monoclonal antibodies that lock the glycoprotein in its pre-fusion conformation were required to stabilize the trimer (19, 21, 25). Notably, these antibodies do not bind across protomers to stabilize trimers. Instead, stabilizing the pre-fusion conformation of the glycoprotein and reducing conformational variability induces the formation of pre-fusion trimers. The IKOV-G pre-fusion trimer, in contrast, was solved via X-ray crystallography in the absence of any stabilizing antibodies, likely aided by stronger binding at the trimeric interface.

In the pre-fusion RABV-G trimer, the interface between protomers consists of three loops in the central domain: the small helix (residues 296–305), the bracket loop (residues 378–388), and the corkscrew loop (residues 259–271) (19). A series of hydrogen bonds between these loops stabilizes the trimer. While the central domain also contains an α-helix at the core of the glycoprotein trimer, the helices are too far apart in the trimer’s protomers to interact and form a trimeric interface (19, 20). In addition to the interactions in the central domain, the conserved aromatic amino acids in the fusion loops also play a role in forming the RABV-G pre-fusion trimer (18, 19). Substitution of these amino acids causes RABV-G to form monomers, even in combination with pre-fusion stabilizing monoclonal antibodies that would otherwise facilitate trimerization (19).

## STRUCTURES OF OTHER LYSSAVIRUS GLYCOPROTEINS: IKOMA AND MOKOLA GLYCOPROTEINS

Despite sharing only ~50% amino acid sequence identity (15), IKOV-G pre-fusion trimers have a remarkably similar structure to those of RABV-G. The overlaid structures have an RMSD of 2–2.4 Å, and the IKOV-G trimeric interface is similar to that of RABV-G but forms more extensive protomer-protomer contacts (Fig. 1B) (23). Notably, IKOV-G crystallizes as a pre-fusion trimer without the addition of stabilizing antibodies and even when the fusion loops are replaced with glycine-serine linkers (23). The post-fusion Mokola virus glycoprotein (MOKV-G) monomer, sharing ~50% sequence identity to RABV-G and ~50% to IKOV-G (15), has been determined via X-ray crystallography (22, 23). As with RABV-G, post-fusion MOKV-G contains an elongated α-helix in the central domain, and the overlaid structures of RABV-G and MOKV-G are strikingly similar (Fig. 1C).

## THERAPEUTIC MONOCLONAL ANTIBODY COCKTAILS TO REPLACE HUMAN RABIES IMMUNOGLOBULIN

Another recent focus in the field has been on using glycoprotein-antibody structures to develop therapeutic monoclonal antibody cocktails as a replacement for human rabies immunoglobulin (HRIG). HRIG consists of polyclonal serum derived from vaccinated humans and, in combination with a rabies vaccine series, is a crucial component of rabies post-exposure treatment. After rabies exposure, HRIG is administered at the wound site to neutralize lingering virus, improving the efficacy of treatment to nearly 100% survival rates (40). However, despite its efficacy in rabies treatment, HRIG has much room for improvement. First, because it is derived directly from human blood, HRIG is very expensive (costing 700 USD on average in 2004 [14]), and replacement with synthetic antibodies could substantially lower the cost of treatment. Second, polyclonal serum is inherently heterogeneous. While formulated to contain a minimum fixed titer of rabies neutralizing antibodies (41), HRIG antibodies in different lots may vary in where they bind, how broadly they neutralize, and how they neutralize. This variability is less than ideal in developing pan-lyssavirus therapeutics. Finally, monoclonal antibody therapies offer increased safety over human blood products, eliminating much of the danger of transmission of undetected blood-borne pathogens. As of this writing, HRIG remains the standard of care in much of the world, but several promising therapeutic antibody cocktails are under development (4, 5, 42–44).

One particularly exciting candidate to replace HRIG was reported to treat symptomatic rabies in mice and consists of two monoclonal antibodies: RVC20 and RVC58 (4, 6). In the study, the authors administered the antibodies through both intramuscular and intracerebroventricular injections, the latter of which delivered antibodies to the cerebrospinal fluid. When administered on the day of symptom onset, the treatment was effective in clearing infection in approximately 30% of mice (4). (Symptomatic infection is otherwise 100% lethal.) These results indicate that antiviral therapeutics for symptomatic patients will need to cross the blood-brain barrier to be effective and that therapeutic antibodies can be developed as such treatments.

## HIGH-RESOLUTION STRUCTURES ILLUMINATE RABIES ANTIGENIC SITES

**Fig 2:**
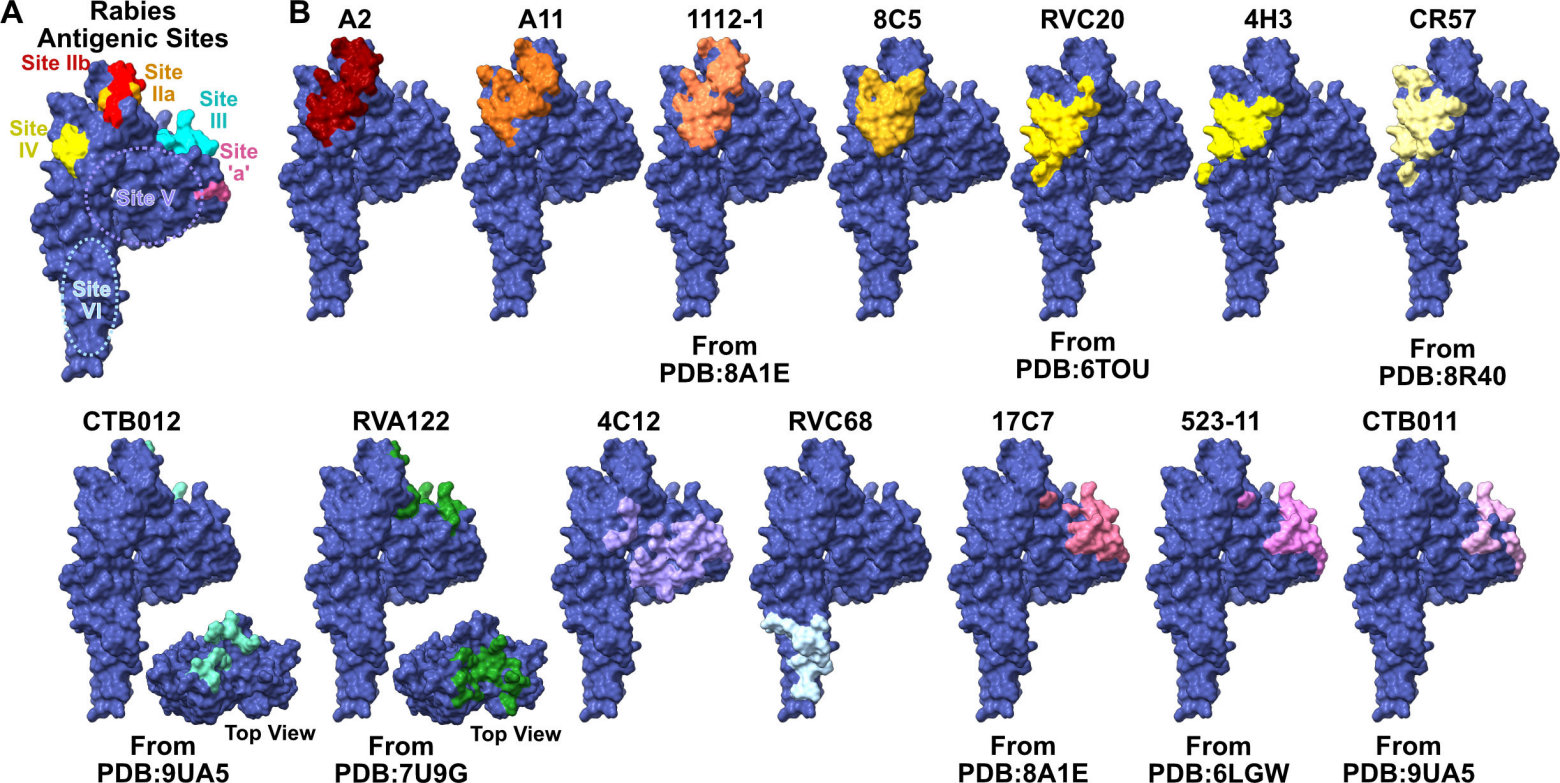
Rabies antibody binding spans the surface of the glycoprotein. (**A**) Rabies antigenic sites IIa, IIb, III, IV, and ‘a’, as defined by the mutagenesis-based naming scheme, plus newly identified antigenic sites V and VI. Note that antigenic site I is not visible from this perspective. (**B**) Rabies antibody binding footprints from high-resolution protein structures, grouped by antigenic site. Footprints depict amino acids within 4 Å of the antibody, mapped onto the RABV-G monomer from the RABV-G/RVC68 co-structure, which has the most complete fusion domain.

Since 2020, multiple structures of RABV-G in complex with neutralizing monoclonal antibodies (18–21, 24, 25) (Fig. 2, Table 1) have been solved, improving understanding of rabies conformations and antibody binding, and facilitating efforts to develop a monoclonal antibody cocktail to replace HRIG. Prior to this work, understanding of rabies antibody binding was limited to information gathered through competition assays and mutagenesis assays (15, 26–28). These new structures have given substantial insight into our understanding of how antibodies recognize and neutralize rabies virus.

**TABLE 1:** Summary of high-resolution structures of RABV-G in complex with neutralizing monoclonal antibodies

mAb	Epitope	Conformation bound	Known viral recognition^*a*^	mAb footprint (residues within 4 Å)^*b*^	Known escape mutations^*c*^
A2 (25)	Sites II/IV	Pre-/post-fusion (25)	RABV (25)	36–44, 189, 192, 194, 196, 198, 199, 203-204, 228, 230-231, 242–244	
A11 (25)	Sites II/IV	Pre-/post-fusion (25)	RABV (25)	39, 41–44, 147, 187, 189, 194, 196, 199, 201, 204, 206, 226, 228–231, 242-244, 246, 248, 251	
1112-1 (20)	Sites II/IV	Pre-/post-fusion (20)	RABV (20)	36–42, 44, 189, 192, 194, 196, 198, 199, 202, 203, 228–231, 242–245	
8C5 (25)	Sites II/IV	Pre-/post-fusion (25)	RABV, DUVV, EBLV-1(25)	42–44, 47, 188–190, 192–196, 228–231, 242–245	
RVC20 (24)	Sites II/IV	Pre-fusion only; epitope exists in post-fusion, but mAb clashes with fusion domain and antibody does not bind at pH 5.6 (20, 24)	RABV, DUVV, EBLV-1, EBLV-2, ABLV, IRKV, KHUV, ARAV, SHIBV, IKOV, BBLV (6)	42, 44, 47, 186–192, 194, 226–231, 251	K226E/N, G229E (6); M44, R147, K226, G229, V230, A242 (45)
4H3 (25)	Sites II/IV	Pre-/post-fusion (25)	RABV, DUVV, EBLV-1 (25)	44, 144–147, 164, 187, 189, 190, 192, 194, 226, 228–231, 251	
CR57 (46)	Site II/IV	Pre-fusion only (46); epitope exists in post-fusion, but mAb clashes with fusion domain	RABV, DUVV, EBLV-2, ABLV, IRKV, KHUV, ARAV (6)	44, 187–189, 192, 194, 224, 226, 228–231, 242, 244, 246, 251, 252, 254	K1, G34, M44, R147, D190, K198, K226, G229, V230, A242, Q382 (45); K226N/E, G229E (47)
CTB012 (21)	Site III	Pre-fusion only (21); epitope does not exist in the post-fusion conformation	RABV (21)	1, 28, 29, 31, 33, 34, 267–272, 275	F2, N27, L28, V29, E31, D32, E33, G34, Y50, I51, K198, A200, V210, I268, E269, H270, L271, V272, E274, E275, L276, V308, F311, D326 (45)
RVA122 (19)	Site III	Pre-fusion only (19); epitope does not exist in the post-fusion conformation	ABV, DUVV, EBLV-1, EBLV-2, ABLV, IRKV, KHUV, ARAV (6)	1–3, 29, 31, 33, 35, 36, 38, 198, 213–216, 270, 271, 309, 331, 333, 334, 337	P137S/R333Q (19); K1, V29, E31, L38, K198, A200, V210, P309, S331, R333, T334, N336, E337, I338 (45)
4C12 (25)	Site V	Pre-fusion only (25); epitope does not exist in the post-fusion conformation	RABV, DUVV, EBLV-1, IRKV (25)	10, 11, 13-16, 47, 190, 193, 194, 318, 319, 340–344, 346, 348–355	
RVC68 (25)	Site VI	Pre-/post-fusion (25)	RABV, DUVV, EBLV-1, EBLV-2, ABLV, IRKV, KHUV, ARAV, LBV, MOKV, SHIBV (6)	90, 92, 93, 96, 97, 100, 103, 105–107, 110, 111, 113-116, 118, 129, 132	A97, W100, D105, R107, E110, L132 (45)
17C7 (20)	Site ‘a’	Pre-/post-fusion (20)	RABV (20)	333, 334, 336, 337, 340–342, 344, 346, 349, 350, 368, 370, 371, 376, 380	R333, T334, N336, E337, I338, P340, S341, R346 (45)
523-11 (18)	Site ‘a’	Pre-/post-fusion (18)	RABV (18)	212, 333, 334, 336, 337, 340–342, 344, 346, 348, 349, 351, 370, 380, 382	
CTB011 (21)	Site ‘a’	Pre-fusion only (21); epitope exists in the post-fusion conformation, but CTB011 has been reported to block the post-fusion transition (21)	RABV (21)	334, 336, 337, 339, 341, 342, 349, 368, 370, 371	

^*a*^ Viral recognition includes a list of viruses that the antibody neutralizes or whose glycoproteins are reported to bind the antibody. Not all antibodies have been extensively tested for cross-reactivity. RABV, rabies virus; DUVV, Duvenhage virus; EBLV-1, European Bat Lyssavirus 1; EBLV-2, Eastern Bat Lyssavirus 2; ABLV, Australian Bat Lyssavirus; IRKV, Irkut lyssavirus; KHUV, Khujand virus; ARAV, Aravan virus; LBV, Lagos Bat virus; MOKV, Mokola lyssavirus; SHIBV, Shimoni Bat virus; BBLV, Bokeloh bat lyssavirus.

^*b*^ The first 19 amino acids of RABV-G are a signal peptide that is proteolytically cleaved and is not present in the mature glycoprotein. Antibody contacts are numbered on the mature glycoprotein, not including the cleaved signal peptide (i.e. Lys1, instead of Lys20). This numbering scheme follows standard conventions from the literature.

^*c*^ Not all antibodies with structures have been tested for escape mutations.

Seven antigenic sites have thus far been identified on RABV-G, spanning the surface of the glycoprotein (Fig. 2), with more likely undiscovered. The antigenic sites are numbered I–VI and ‘a’, corresponding to a glycoprotein mutagenesis-based naming scheme in which antibodies were grouped into epitopes according to competition assays and the point mutations disrupted binding (26–28). Those mutations correspond to residues 263–264 (site I), 198–200 and 34–42 (site IIa and IIb, respectively), 330–338 (site III), 226–231 (site IV), and 342–343 (site ‘a’) (15). Antigenic sites V and VI were recently identified (25) and build on this mutagenesis-based naming scheme.

Site I is located on the side of the central domain and no structures of site I antibodies in complex with RABV-G have yet been determined. Antigenic sites II and IV comprise an overlapping region on the Pleckstrin homology domain (Fig. 2) (25) and are one of two major antigenic sites on RABV-G (6, 27). The Pleckstrin homology domain and sites II/IV maintain a nearly identical conformation in both the pre-fusion and post-fusion states (18, 19, 24). Antibodies that recognize sites II/IV bind both pre- and post-fusion conformations of RABV-G (25) and do not stabilize either glycoprotein conformation. Eight structures of site II/IV antibodies have been determined thus far, including antibodies A2 (25), A11 (25), 1112-1 (20), 8C5 (25, 48), RVC20 (6, 24), 4H3 (25, 48)**,** 7E8 (25, 48), and CR57 (46). The combined structures show that antigenic sites II and IV form a continuum across the Pleckstrin homology domain. While some antibodies bind at opposite ends of the continuum and may be considered strictly site II or site IV antibodies, others recognize both sites (20, 25), indicating that antibody binding to the Pleckstrin homology domain is more nuanced than indicated in the initial mutagenesis-based studies.

Unlike antigenic site II/IV, antibodies targeting site III, the second major antigenic site, are specific to pre-fusion RABV-G. When RABV-G transitions into the post-fusion conformation, the Pleckstrin homology domain sits on top of the central domain and blocks access to antigenic site III (18–20) (Fig. 1). Antibodies that recognize antigenic site III may either stabilize pre-fusion RABV-G when they bind, in turn stabilizing the pre-fusion trimer, or bind pre-fusion RABV-G without facilitating trimerization. Trimer-stabilizing site III antibodies include RVA122 and CTB012 (Fig. 2) (6, 19, 21), which aid structural determination of the RABV-G pre-fusion trimer, while non-stabilizing site III antibodies include 10H5 (25, 48) and A4 (25).

Three RABV-G-antibody structures have been determined at site ‘a’: one in complex with the post-fusion monomer (antibody 623–11) (18), and two in complex with the pre-fusion trimer (antibodies 17C7 [20] and CTB011 [21] [Fig. 2]). Antigenic site ‘a’ is present in both pre-fusion and post-fusion conformations of RABV-G, and in the three structures of antibodies that recognize this site, the antibody binding footprint is constrained to the central domain (Fig. 2; 18, 20, 21).

Recently, two new antigenic sites were identified in high-resolution cryo-EM structures (25), designated sites V and VI, in a continuation of the classic mutagenesis naming scheme. Site V is located on the side of the central domain below antigenic site ‘a’ (Fig. 2A) (25), while site VI is located on the fusion domain (Fig. 2) (6, 25, 45). Site V antibody 4C12 is pre-fusion specific, linking the pre-fusion central domain to the Pleckstrin homology domain (25). In contrast to antibody 4C12, site VI antibody RVC68 binds solely to the fusion domain and recognizes the full range of glycoprotein conformations (6, 25, 45). Like the Pleckstrin homology domain, the fusion domain maintains the same overall conformation in the pre-fusion and post-fusion states (Fig. 1) (18).

Site V antibody 4C12 and especially site VI antibody RVC68 are also notable for their broad recognition of both RABV-G and other lyssavirus glycoproteins, including those from Irkut, Duvenhage, Ikoma, Mokola, and/or Eastern bat lyssavirus 1 lyssaviruses (6, 25). This broad glycoprotein recognition is driven by high amino acid sequence conservation at antigenic sites V and VI, greatly exceeding that of the rest of the glycoprotein (25). These newly identified antigenic sites may be particularly good targets for pan-lyssavirus antibodies in a therapeutic cocktail or for emphasis in a pan-lyssavirus vaccine.

## RESISTANCE AND ESCAPE MUTATIONS TO MONOCLONAL ANTIBODIES

In transitioning from polyclonal antibody therapy (HRIG) to a monoclonal antibody cocktail, viral escape mutations that confer resistance to the components of the cocktail are a major concern. Some of these mutations have been determined for individual monoclonal antibodies (6, 28, 36), and others can be predicted from high-resolution protein structures (18–21, 24, 25) or from grouping antibodies into the classical rabies antigenic sites that were defined by mutations conferring antibody resistance (6, 15). However, a recent study using deep mutational scanning has explored rabies antibody escape mutations for therapeutic antibody candidates in much greater depth (45). In the study, the authors constructed a DNA library containing RABV-G with every possible individual amino acid mutation, generated pseudoviruses, and then determined which pseudoviruses were resistant to antibodies (45).

One particularly interesting finding from the study was that while for some antibodies, there was a wide array of escape mutations that spanned most of the binding footprint, for other antibodies, escape mutations were concentrated into much smaller sites of 2–3 amino acids. For these antibodies, which included RVC20, RVA122, and RVC68 (45), it may be possible to engineer resistance to viral escape mutations using the high-resolution structures that have already been determined (19, 24, 25).

## ANTIBODY TARGETS AND VARIATION IN THE RABIES POLYCLONAL ANTIBODY RESPONSE

In engineering improved rabies vaccines, three major areas of focus are improving the duration, potency, and breadth of the neutralizing antibody response. Modern rabies vaccines for human and veterinary use consist of inactivated rabies virus that is either unadjuvanted (human) or adjuvanted with alum (veterinary) (49, 50). Wildlife rabies vaccines, delivered in edible baits, were historically live-attenuated rabies strains but have since transitioned to vectored vaccines (51) because of the danger of reversion to more virulent forms of rabies. However, each of these vaccine types displays wild-type RABV-G and induces an antibody response that can vary substantially between individuals (3, 15, 52).

Most rabies vaccine antibody responses are both short-lived and rabies-specific, with little breadth of neutralization (3, 6, 53). However, there have been reports of rarer individuals with long-lasting, more potently neutralizing, and/or more broadly neutralizing antibodies or antibody responses. Broadly neutralizing monoclonal antibodies identified in reference 6 provide one such example. Another study in human rabies immunization showed that some individuals produced neutralizing antibody titers that were 2- to 3-fold higher than the mean (over 10-fold higher than the lowest titers), and others kept high neutralizing antibody titers after 5 years instead of dropping to undetectable antibody levels (3).

One possibility is that these antibodies target uncommon epitopes or conformations of RABV-G. Examples of such epitopes include antigenic sites V and VI (25), where residues are more highly conserved than the rest of the glycoprotein. Furthermore, longer-lasting antibody responses may also be linked to epitopes on RABV-G, as substantial differences in the duration of the neutralizing antibody response were also observed in genetically identical animals (54). Identifying, stabilizing, and emphasizing the epitopes that induce broadly neutralizing, potently neutralizing, and long-lasting antibody responses will likely prove critical for the development of an improved pan-lyssavirus vaccine.

## RABIES RECEPTORS

Just as important as understanding antibody binding in rabies and related lyssaviruses is determining what receptor(s) these viruses use to drive infection. Viral receptors give crucial insight into viral host range and transmission, allowing predictions of new host species that may be susceptible to infection. The viral receptor binding site is also a potential target for both antiviral drug development and vaccine design. Currently, potential receptors have only been identified for rabies virus, with little understanding of how non-rabies lyssaviruses enter cells or whether they use receptors distinct from those of rabies virus. Furthermore, the proteins or other molecules that rabies uses as receptor(s) and/or attachment factor(s) are still an active area of research.

To date, five proteins and two types of non-protein ligands have been reported to promote rabies cellular attachment and infection, supported by varying levels of evidence. These potential receptors and attachment factors include the nicotinic acetylcholine receptor (nAChR), the p75 neurotrophin receptor (p75NTR), the neuronal cell adhesion molecule 1 (NCAM1), the metabotropic glutamate receptor 2 (mGluR2), integrin β1, heparan sulfate proteoglycan (HSPG), and phosphatidylserine (55–60). As the only protein on the surface of the virus, RABV-G is also responsible for mediating attachment to cells. However, given the relatively small surface area on RABV-G available for receptor binding and the lack of other surface glycoproteins to mediate contact with a receptor, it seems unlikely that RABV-G would form specific interactions with each proposed ligand. An added complication of investigating rabies receptor binding is that no non-permissive mammalian cell line has yet been identified for rabies, making it difficult or impossible to run the classic experiment of expressing a potential receptor on a non-permissive cell line and determining if that receptor enables infection. However, despite these complications, there is compelling evidence that these proteins do impact rabies infection.

## THE NICOTINIC ACETYLCHOLINE RECEPTOR (nAChR)

**Fig 3:**
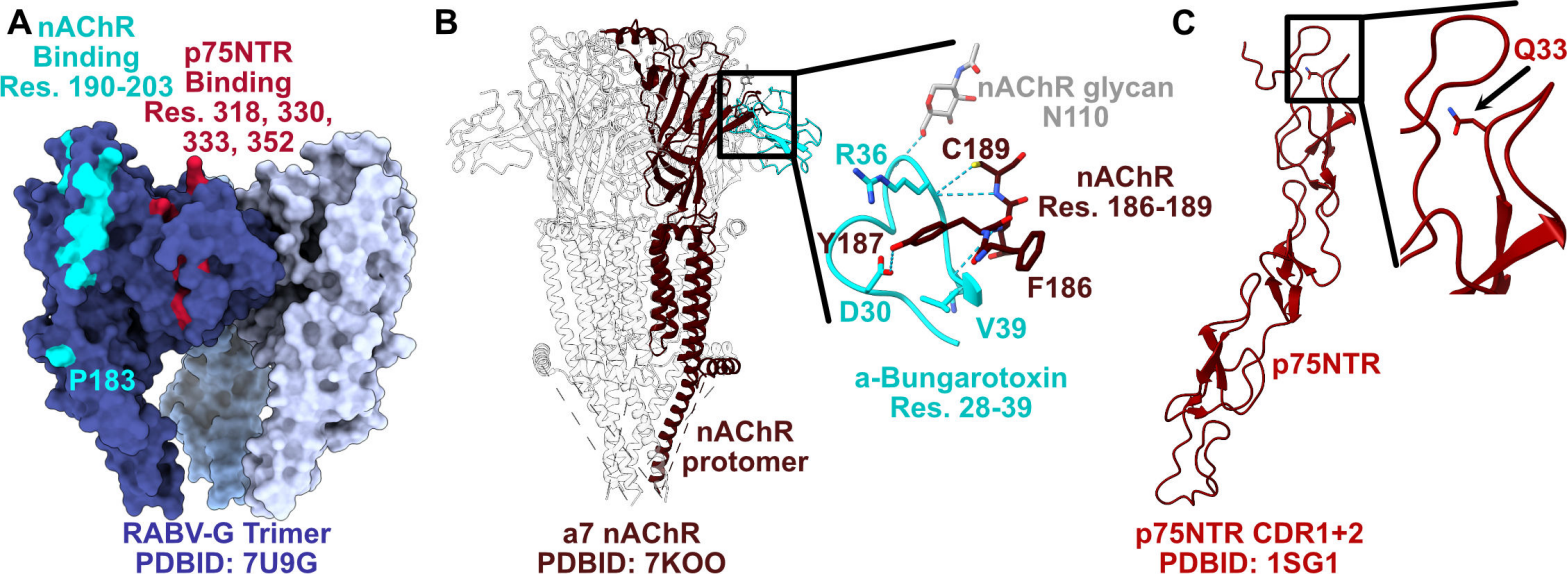
Potential rabies receptor binding sites are in the RABV-G Pleckstrin homology domain and central domain**.** (**A**) Potential binding sites for the nAChR and the p75NTR, mapped onto the RABV-G pre-fusion trimer. Residue P183 is highlighted. (**B**) The α7 nAChR bound to α-bungarotoxin, with the region of sequence homology between RABV-G and α-bungarotoxin displayed in detail. (**C**) The p75NTR with predicted RABV-G binding residue Q33 highlighted.

The nicotinic acetylcholine receptor (nAChR) is a pentameric, ligand-gated membrane transporter that controls the movement of calcium into neurons (61) and currently has the strongest evidence for involvement in rabies infection and disease. First, the nAChR is present in both neuromuscular junctions and synapses in different isoforms (62, 63), placing it on both types of neurons that rabies infects in a host: motor neurons and neurons of the central nervous system. Second, RABV-G also shares amino acid sequence identity with the nAChR ligand α-bungarotoxin (64), and nAChR peptides corresponding to residues 174–203 both bind to rabies virus and inhibit viral infection (65). Furthermore, studies tracking the evolution of rabies when it adapts to new host species have found evidence of positive selection at RABV-G residue 183 (66), located adjacent to the loop predicted to interact with the nAChR (RABV-G residues 190–203, Fig. 3A) (64, 67). Finally, the addition of RABV-G to cells expressing the nAChR causes changes in neuronal signaling. When human nAChR is expressed on frog oocytes, addition of RABV-G peptides reduces signaling across the cell membrane (68). Similarly, when RABV-G peptides are injected into the brains of mice, the mice exhibit behavioral changes, including increased locomotion, that are consistent with symptoms of rabies disease (69).

The nAChR exists as multiple isoforms and may be either a homopentamer composed of identical subunits or a wide variety of different heteropentamers (62). For rabies infection, perhaps the most relevant nAChR isoforms are the α7 homopentamer, present on neurons of the central nervous system, and α1-containing heteropentamers, found on motor neurons (63, 68). In 2021, the first structures of the human α7 nAChR homopentamer were determined via cryo-EM, both alone and in complex with α-bungarotoxin (61), giving further insight into the potential interaction between RABV-G and the nAChR and changes in nAChR signaling. In these structures, the nAChR region associated with rabies binding is an outward-facing loop on the extracellular domain (Fig. 3B; 61). Depending on the bound ligand, the ion channel in the center of the nAChR may be in either the open (active), closed (resting), or desensitized conformation (61). When α-bungarotoxin binds the nAChR, the ion channel enters a resting state, where it narrows to prevent the flow of calcium ions (61). If RABV-G binding to the nAChR also causes a conformational change that closes the ion channel, it would be expected to impact neuronal signaling.

While experimental evidence that the nAChR is involved in rabies disease is strong, whether it functions as a receptor, attachment factor, or plays another role in infection remains to be determined. There is evidence that the nAChR undergoes endocytosis (70), making it possible that the nAChR is a true receptor for rabies, both binding to viral particles and facilitating their uptake into cells. However, an alternative explanation for the experimental evidence above is that rabies interactions with the nAChR are important not for cellular uptake but for inducing behavioral changes in the host. Such changes, including aggression and increased locomotion that drive infection, are crucial for organism-to-organism spread (69, 71). Another important caveat is that RABV-G residues 190–203 adopt a different conformation than the corresponding residues from α-bungarotoxin (Fig. 3), raising questions about whether this part of the glycoprotein interacts with the nAChR. Determining whether the nAChR functions as a rabies receptor will require expressing the nAChR on a truly rabies-resistant mammalian cell line, a resource that has not yet been developed, and observing if the addition of the nAChR permits infection.

## THE p75 NEUROTROPHIN RECEPTOR (p75NTR)

Aside from the nAChR, the protein with the best evidence of functioning as a rabies receptor or attachment factor is the p75 neurotrophin receptor (p75NTR). The p75NTR is a single-pass, monomeric transmembrane protein that contains multiple discrete cysteine-rich domains (CRDs) and is also found on neurons of the central nervous system but not motor neurons (55). Known ligands for the p75NTR include neurotrophins such as nerve growth factor, as well as the glycosphingolipid ganglioside GT1b and the amyloid β peptide associated with Alzheimer’s disease (55).

The strongest evidence in support of the p75NTR as a rabies receptor is a series of binding and infection inhibition studies with full-length and soluble p75NTR (72–74). While the lack of cell lines non-permissive to rabies infections makes these studies challenging, cell lines transfected to overexpress the p75NTR have substantially more binding to RABV-G (72, 73). Furthermore, when the p75NTR is expressed as a soluble dimer via fusion to an antibody Fc domain, it inhibits rabies infection in a dose-dependent manner (74). Specific point mutations to RABV-G at residues 318 and 352 inhibited this p75NTR-Fc-mediated viral neutralization and binding to the p75NTR (74). However, viruses containing these mutations were still able to grow on BSR cells, albeit at reduced titers (74), indicating that while the p75NTR may aid in rabies infection, it may not be essential.

On the p75NTR, constructs with deletions of individual CRDs indicate that the most N-terminal CRD, CRD1, is responsible for binding to RABV-G (Fig. 3C; 73). Point mutations to CRD1 further narrowed the RABV-G binding site to a region surrounding p75NTR residue Q33 (73). A crystal structure of the first two p75NTR CRDs from the human protein (75) reveals that Q33 is located close to the protein’s N-terminus and at the outermost-facing edge of CRD1 (Fig. 3C). Overall, the existing evidence best supports the p75NTR as a rabies attachment factor capable of enhancing rabies infection but ultimately not essential for it.

## THE NEURONAL CELL ADHESION MOLECULE (NCAM1)

The final classically proposed rabies receptor is the neuronal cell adhesion molecule (NCAM1). Like the p75NTR, NCAM1 is a single-pass transmembrane protein expressed on neuronal cells (76), and like the nAChR, it exists as multiple different isoforms. Each NCAM1 isoform has a cytoplasmic domain of a different length (NCAM120, 140, and 180, listed from shortest to longest) but an identical extracellular domain (55, 76). The NCAM1 extracellular domain consists of five immunoglobulin (Ig)-like domains followed by two fibronectin-like domains (76). The first three Ig-like domains and the two fibronectin-like domains have been solved via X-ray crystallography, giving insight into the overall shape of the protein (77, 78).

Current evidence in support of NCAM1 as a rabies receptor or attachment factor includes observations that NCAM1 is located in neuronal synapses (76, 79, 80) and that cells that lack NCAM1 on their surface are less susceptible to rabies infection (55). Furthermore, NCAM1 knock-out increases survival time in rabies-infected mice and decreases the number of infected cells in their brains (79). Perhaps most interesting, however, was the observation that the addition of heparan sulfate, a ligand for NCAM1 (76), substantially decreases rabies infection in cells, but the structurally similar polysaccharide chondroitin sulfate does not (79). This finding suggests that RABV-G may interact with NCAM1 via heparin-sugar binding (described below) rather than forming a direct protein-protein interaction with NCAM1 itself. Overall, this evidence supports NCAM1 functioning as an attachment factor for rabies but not necessarily a receptor, as NCAM1 appears to be non-essential for infection.

## NEW REPORTS OF RABIES RECEPTORS: mGluR2 AND INTEGRIN β1

More recently, with the development of siRNA knock-down screens, there have been studies reporting additional possible receptors for rabies virus, including the metabotropic glutamate receptor 2 (56) and integrin β1 (57). As more recent findings, comparatively less information is currently available about precisely how these proteins are involved in rabies infection.

The metabotropic glutamate receptor 2 (mGluR2) was identified in an siRNA knock-down screen and, when expressed as a soluble ectodomain, was reported to inhibit rabies infection in tissue culture and increase survival of rabies-infected mice (56). While the authors also report a protein-protein interaction between the mGluR2 and RABV-G ectodomains via co-precipitation assays, one important caveat to the finding is that binding was reported when both proteins were expressed in *E. coli* and lacked glycans. As glycosylation is essential for proper folding and secretion of RABV-G (81), it remains unclear how RABV-G and the mGluR2 interact.

In a similar experimental approach, the same group identified integrin β1 as an entry factor and an additional potential receptor for rabies virus (57). As with their study on the mGluR2, the authors report that soluble integrin β1 ectodomains inhibit rabies infection in tissue culture, increase survival of rabies-infected mice, and directly pull-down RABV-G ectodomains (57). However, the same caveats regarding *E. coli*-expressed RABV-G apply to the pull-down assays, and it also remains unclear how RABV-G and integrin β1 interact. One additional interesting report from the study, however, was that integrin β1 and the nAChR interact (57), which could explain why both soluble integrin β1 and antibodies against integrin β1 were reported to reduce rabies deaths in mice. As an alternative explanation, integrin β1 has been reported to bind to fibronectin (82), which in turn binds to heparin (83). As with NCAM1 described above, if RABV-G interacts with integrin β1 indirectly through heparin, it may explain these results.

## NON-PROTEIN LIGANDS FOR RABV-G (SUGARS AND LIPIDS)

In addition to protein receptors/attachment factors, RABV-G has also been reported to bind to sugars and lipids (19, 55, 58–60, 84–86), which may explain why rabies infects such a broad range of species and cells. However, it is also important to note that similar reports of lipid binding were made for the rhabdovirus VSV (87), but it was subsequently determined that the lipid does not function as a viral receptor (88). Whether this finding is also true for rabies infection remains to be determined.

Specific molecules reported to bind to rabies include phosphatidylserine, phosphatidylethanolamine, and phosphatidylinositol (58, 59), as well as sialic acid, galactose, mannose, N-acetyl-glucosamine, and heparin (60, 84, 85). RABV-G lipid binding interactions are controlled by the viral fusion loops, with residue W121 being the most important for binding (19). Notably, soluble RABV-G ectodomains with unmodified fusion loops co-purify with cellular lipid membranes (19), while those with the fusion loops replaced with a glycine-serine linker or with a W121A point mutation do not (18, 19).

The precise role that glycoprotein binding to non-protein ligands plays in rabies infection requires additional study but may involve cellular attachment or stabilization of the viral glycoprotein. On rabies virions, some proportion of glycoproteins remain in the post-fusion conformation even at neutral pH (32), with their fusion loops pointed away from the viral membrane. Through avidity binding interactions, several post-fusion glycoproteins bound to cellular lipids or glycans simultaneously could help attach a virus to a cell surface. While the virus would still need to undergo endocytosis and likely bind to a specific receptor to facilitate it, the initial cellular attachment could allow rabies to use cellular receptors it has relatively low binding affinity for. Lipid binding also stabilizes the pre-fusion conformation of RABV-G, with mutations to aromatic fusion loop residues, especially W121, decreasing the proportion of glycoproteins in the pre-fusion conformation (19). When its fusion loops are bound to a lipid membrane, pre-fusion RABV-G trimers must unbind both from the membrane and from the other subunits of the trimer to transition to the post-fusion conformation (19). Stabilizing the pre-fusion conformation may in turn help to ensure that a receptor binding site remains accessible on a proportion of the glycoproteins or to prevent too many glycoproteins from transitioning to the post-fusion conformation.

## THREE HYPOTHESES FOR RABV-G RECEPTOR AND LIGAND BINDING INTERACTIONS

RABV-G is unlikely to form unique, direct protein-protein interactions with so many different receptors. The potential receptors share no notable sequence homology, and RABV-G simply lacks the surface area for five or more distinct receptor binding sites. Instead, three possibilities are much more likely. For the first possibility, rabies has a main protein receptor that facilitates both attachment and uptake into neuronal cells during infection. This receptor would need to be present on both motor neurons and neurons of the central nervous system, be highly conserved, and undergo endocytosis. Of the potential receptors identified so far, the nAChR best fits these requirements. The remaining RABV-G ligands could serve as attachment factors or modulate nAChR expression/location, thereby impacting infection.

A second possibility is that rabies would first attach to cells through non-protein interactions with sugars or lipids, either present on the membrane or associated with another protein. After attaching to cells, the virus would then need to bind a protein undergoing endocytosis for uptake into the cell. The protein facilitating endocytosis could either interact directly with RABV-G or interact indirectly via a bound sugar or lipid.

For the third possibility, RABV-G could recognize a conserved protein fold, such as a CRD, fibronectin-like domain, or immunoglobulin-like (Ig) domain. Through multiple lower-affinity binding interactions with this fold, rabies virions could attach to cells. If the protein containing the conserved fold undergoes endocytosis, it could also facilitate viral entry into the cell. Alternatively, once attached to the cell, RABV-G could bind to a second protein to facilitate endocytosis.

Testing these hypotheses and determining precisely how rabies binds to and enters cells will prove challenging. The first will require a completely non-permissive mammalian cell line to test potential receptors. The second will require eliminating both RABV-G sugar and lipid binding, which will likely disrupt the viral fusion loops and render the virus non-infectious. Finally, the third would be best supported by a high-resolution structure of RABV-G in complex with the conserved fold.

## FUTURE DIRECTIONS

The newly available lyssavirus glycoprotein structures and structures of RABV-G in complex with neutralizing antibodies represent substantial progress in the structure-guided design of improved rabies vaccines and therapeutics. However, our understanding of lyssavirus glycoprotein structure and function remains incomplete. Of the 18 known lyssaviruses, glycoprotein structures have only been determined for three (rabies, Ikoma, and Mokola), and no structure of the post-fusion trimer has been determined for any lyssavirus. While the overall shapes of lyssavirus glycoproteins are expected to be similar, glycoproteins from different lyssaviruses may differ in the stability of their conformations or in the epitopes emphasized during vaccination. Furthermore, where antibodies from long-lasting rabies immune responses bind RABV-G and the factors that lead to these long-lasting responses remain unclear. Finally, no structures of RABV-G in complex with receptors or attachment factors have yet been determined, and the proteins or molecules that rabies uses as cellular receptors are still uncertain.
